# Chitinase-producing bacteria and their role in biocontrol

**DOI:** 10.3934/microbiol.2017.3.689

**Published:** 2017-08-04

**Authors:** Esteban A. Veliz, Pilar Martínez-Hidalgo, Ann M. Hirsch

**Affiliations:** 1 Department of Molecular Cell and Developmental Biology, Molecular Biology Institute, University of California, Los Angeles, 90095-1606, USA; 2 Departamento de Microbiología y Genética, Universidad de Salamanca, Salamanca, Spain

**Keywords:** chitin, chitinases, plant-microbe interactions, inoculant, biocontrol agent, biopesticide, postharvest

## Abstract

Chitin is an important component of the exteriors of insects and fungi. Upon degradation of chitin by a number of organisms, severe damage and even death may occur in pathogens and pests whose external surfaces contain this polymer. Currently, chemical fungicides and insecticides are the major means of controlling these disease-causing agents. However, due to the potential harm that these chemicals cause to the environment and to human and animal health, new strategies are being developed to replace or reduce the use of fungal- and pest-killing compounds in agriculture. In this context, chitinolytic microorganisms are likely to play an important role as biocontrol agents and pathogen antagonists and may also function in the control of postharvest rot. In this review, we discuss the literature concerning chitin and the basic knowledge of chitin-degrading enzymes, and also describe the biocontrol effects of chitinolytic microorganisms and their potential use as more sustainable pesticides and fungicides in the field.

## 1. Introduction

Chitin in nature is both abundant and widespread. Indeed, it is one of the most abundant biopolymers on Earth, second only to cellulose. Chitin is found in many organisms, including the shells, exoskeletons, and gut linings of arthropods (crustaceans and insects). It also comprises the cell walls of many fungi, including some yeasts, and makes up the structural frameworks of certain Protista as well as of nematode eggs. Many microbial genomes possess different genes encoding chitinolytic enzymes, which have been extensively investigated, but studies regarding the use of microorganisms that utilize insoluble chitin as a carbon source in the area of biotechnology are sparse [1].

According to previous studies, approximately 35% of crop yields are lost to diseases in the field, and postharvest losses average closer to 15% of the total yields [2]. The main causes of the losses are insects, weeds, and diseases. Consequently, crops worldwide are completely dependent on the use of fungicides and pesticides to reduce loss, but the major problem of using these chemicals is that the target organisms often develop resistance to them. Management strategies can mitigate this effect, but they by no means completely prevent resistance from evolving [3]. The impact of pesticides in the environment on non-target organisms, as well as on human health, has been extensively studied with worrying results. Leaching of fungicides into the ground water damages both aquatic environments and drinking water resources [4] and many human health problems have been linked to pesticide use [5]. Recently, a study on honey has found that the samples contained pesticides [6]. Public concern about these chemical residues as well as the development of fungicide resistance by pathogens have prompted the development of alternative approaches to control both pre- and postharvest diseases [3].

Microorganisms produce numerous hydrolytic enzymes, and among them are chitinases. Actinobacteria are well known for their chitinolytic enzyme production and activity like Firmicutes [7] as are certain Proteobacteria [8]. Microbial chitinases weaken and degrade the cell walls of many pests and pathogens, thereby exhibiting antibacterial, anti-fungal, insecticidal, or nematicidal activity [9]. Chitinolytic enzymes will become a more obvious and important solution towards overcoming the environmental and human hazards that result from the application of synthetic pesticides and fungicides. Thus, chitinolytic microorganisms have promise as replacements for the more harmful practices of applying insect- and fungal-killing chemicals.

In this review, we aim to increase the understanding of the role of chitinases in the effect of bacterial inoculants. We analyze the literature on chitin-degrading enzymes in microorganisms that have potential as biocontrol agents, and describe the different uses and modes of action that a microbial inoculant should have against pathogens, not only in the field, but also relative to postharvest storage. We also describe several assays for screening previously under-studied microbial species for chitinolytic activity in order to facilitate their characterization and use in place of pesticides.

## 2. Chitin and Its Hydrolysis

**Figure 1.**
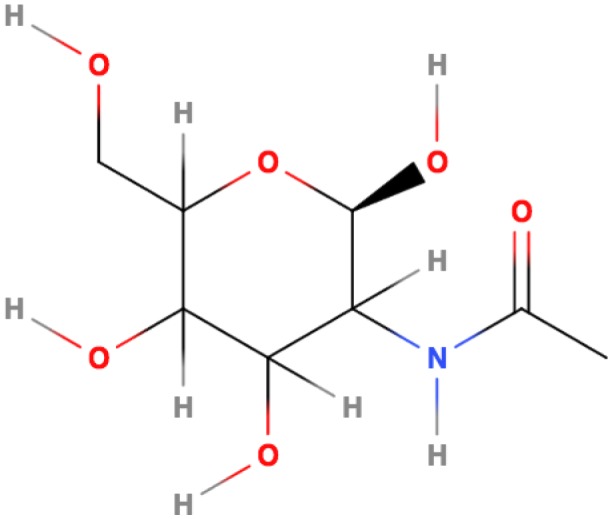
2D structure of N-Acetylglucosamine.

Given the abundance of chitin in nature, the study of its hydrolysis is an interesting topic not only for basic knowledge but also because of the applications of the findings in formulation of inoculants. Chitin is functionally and structurally similar to cellulose (Figure 1). Whereas chitin is a linear homopolymer of (1→4) β-linked N-acetyl-D-glucosamine (GlcNAc) residues, cellulose is composed of glucose monomers. The GlcNAc residues differ from glucose in that an acetyl amino group replaces the hydroxyl at the C-2 position. Chitin is also similar to the structural polymer, murein, which consists of alternating GlcNAc and N-acetylmuramic acid monomers.

Chitin exists in three crystalline forms: α-, β-, and γ-chitin, which differ in the arrangement of polymer chains, giving them different mechanical properties [10]. The different chains cross-link to other structural polymers such as proteins and β-glucans to provide rigidity and strength [11]. In the environment, chitin is found in various degrees of deacetylation, from fully acetylated chitin to its completely deacetylated form, which is known as chitosan [8]. Because of the ubiquity of chitin in the environment, its degradation has been extensively reviewed with regard to general biochemistry, molecular biology, biogeochemistry, and microbial ecology [8],[12],[13],[14].

The degradation of chitin is catalyzed by chitinases, which are found in the organisms containing chitin, mainly insects, crustaceans, and fungi. Moreover these enzymes are also detected in viruses, bacteria, archaea, Protista, higher plants, and animals. Chitinases serve various functions in these organisms, such as in morphogenesis, nutrient cycling, and also in defense against chitin-containing pests and parasites [15],[16]. Chitinolytic bacteria decompose chitin in both aerobic and anaerobic conditions and are found in a wide range of habitats. In marine environments, they are involved in the nutrient cycling of the sizeable amount of chitin derived from arthropod shells and other sources [17]. In the soil and rhizosphere, bacteria use chitin from insects and fungi as a carbon and nitrogen source [13],[18].

Because of the structural similarity of chitin and cellulose, the chitinolytic and cellulolytic pathways follow parallel steps. Chitin hydrolysis consists of first cleaving the polymer into water-soluble oligomers, followed by splitting of these oligomers into dimers by another enzyme, which splits the dimers into monomers. This process involves an endo-acting chitinase (EC 3.2.1.14), which randomly hydrolyzes chitin and the resulting oligomers, releasing a mixture of end products of different sizes. However, this enzyme is unable to break down the molecules beyond diacetylchitobiose. On the other hand, β-N-acetylhexosaminidases (EC 3.2.1.52) are exo-acting, and cleave chitin oligomers and also chitin from the non-reducing end. It is the only enzyme that can cleave diacetylchitobiose. However, the nomenclature for the enzymes currently classified as β-N-acetylhexosaminidases is not completely agreed upon. In the past, the naming system more closely paralleled the classification and the mechanisms used for the cellulolytic enzymes. Now the classification of β-N-acetylhexosaminidase (*nagZ*) encompasses many exo-acting chitinolytic enzymes, which were previously reported to have different affinities, such as those with activity towards small oligomers or long chain polymers [19],[20].

Based on amino acid sequence, chitinases are grouped into different glycoside hydrolase (GH) families, e.g., GH18, GH19, and GH20. GH18 endochitinases are found in bacteria, fungi, and animals; some plant chitinases also fall into this group. Most bacterial chitinases belong to the GH18 family [12]. Bacterial GH18 chitinases are separated into three major subfamilies, A, B, and C, based on amino acid sequence homology of the individual catalytic domains [21]. Each chitinase is thought to have different, albeit complementary mechanisms [22]. Family GH19 endochitinases are primarily found in plants although they are also detected in bacteria, particularly actinobacteria, green non-sulfur (Chloroflexi), and purple (Chromatiales and *Rhodospirillaceae*) bacteria [23],[24],[25]. Plant GH19 chitinases are thought to be part of the defense mechanism against fungal pathogens [23]. Because GH families 18 and 19 do not share sequence similarity, 3D structure, or molecular mechanisms, they are thought to have evolved as separate lineages [15]. Based on amino acid sequence analysis, GH19 chitinases in Actinobacteria are related to plant class IV chitinases. They appear to have originated as plant chitinases, and bacteria may have acquired them through horizontal gene transfer [23],[24]. Several actinobacteria have been reported to contain GH19 chitinases [24], often in combination with GH18 chitinases. Further analysis of gene sequences indicate that an ancestor of the Streptomycineae first acquired a chitinase from plants and later passed it on to other actinobacteria [23]. *Streptomyces*, which has both GH18 and GH19 families, show different chitinolytic efficiencies on different substrates (GH18 on crystalline substrates, and GH19 on soluble substrates), but only the GH19 chitinases have antifungal properties [23]. On the other hand, β-N-acetylhexosaminidases are exo-acting chitinolytic enzymes that remove monosaccharides from the non-reducing ends of chitin [11],[15].

The chitinolytic machinery of bacteria also consists of chitin-binding proteins (CBPs), which with the chitinases [26], work together [27]. Based on the analysis of a variety of genomes, family 33 CBPs are produced by most chitinolytic bacteria [28]. Manjeet et al. [26] described synergisms between GH18 chitinases and chitin-binding proteins from *Bacillus thuringiensis* and *Serratia* *marescens* when tested in combinations with one another, whereas the hydrolytic ability of GH18 chitinases from *Bacillus licheniformis* did not depend on such an interaction. These data agree with the results of Vaaje-Kolstad et al. [28] that CBPs from the same or different organisms can enhance the hydrolytic activity of chitinases on isolated pure substrates.

An alternative pathway involves the deacetylation of chitin to chitosan, which is then broken down to glucosamine residues by chitosanase (EC 3.2.1.132). Chitosanase hydrolyzes β-1,4-glycosidic bonds in polymers that are difficult to break down, turning them into low molecular weight compounds, which are easier to degrade. Chitinases and chitosanases overlap in substrate specificity, but differ in efficiency based on the degree of deacetylation. Cellulases also break down chitosan and bind chitin, but it has not been shown whether these enzymes actually hydrolyze chitin [8].

Glucose and most other hexose and pentose sugars, except arabinose, are inhibitors of chitinase [29],[30],[31]. Other sugars such as pectose and cellulose have been shown to induce a higher expression of chitinases, and divalent cations either inhibit or activate chitinases. Some known divalent cation chitinase inhibitors are Hg^2+^, Cu^2+^, Co^2+^, and Mn^2+^ [30],[32] whereas Mg^2+^, Ba^2+^ and Ca^2+^ are reported to be activators [29],[32].

## 3. Chitinolytic Microorganisms as Sustainable Alternatives to Chemical Pesticides in Pathogen Control

Among the bacteria used as biocontrol agents, the primary ones are species of *Streptomyces*, *Bacillus*, and *Pseudomonas* [33]. Given the number of chitinolytic bacteria being studied, coupled with the fact that the current strategies for pathogen control are harmful, the idea of formulating new pesticides that consist of biocontrol bacteria offer potential solutions. The damaging effects of pesticides on the environment are well established and described in the literature. Not only do these chemicals affect organisms in the ecosystem besides the target organisms, but they also have the ability to move within and outside of the location of application [34]. Moreover, laboratory studies performed by [35] showed a decrease in microbial activity when pesticides were added to the soil in high quantities. In some cases, the microbial activity could not be recovered.

Chitinases have also been demonstrated to affect insect growth; both feeding rate and body weight of larvae decrease if they are in contact with chitinases, which ultimately leads to death. These symptoms are attributed to the weakening of the peritrophic membrane that lines the gut epithelium of the larvae, the main component of which is chitin [36]. Brandt et al. [37] observed that the *Orgyia pseudotsugata* peritrophic membrane was degraded by chitinases and later this effect was also observed in vivo with *Spodoptera littoralis* and *E. coli* that expressed the endochitinase ChiAII from *Serratia marescens* [38].

More recent studies have focused on the search for alternatives to chemical pesticides and *B. thuringiensis* toxin in response to the development of resistance to both agents by insects or pathogenic fungi. To this end, bacteria from different orders have been found to be effective biocontrol agents.

### 3.1. Actinobacteria

Actinobacteria are important saprophytic soil bacteria, which are known for antibiotic and secondary metabolite production, as well as for the synthesis of chitinolytic enzymes [39]. They are among the most important taxa in the soil microbial chitinolytic community [40],[41]. Bai et al. [42] found that almost half of the terrestrial chitinase-containing bacterial genomes they analyzed from public databases belonged to the Actinobacteria.

*Streptomyces* species, which have been thoroughly studied, decompose solid chitin pieces rapidly, in large part because of their ability to penetrate these substrates with their hyphae [42],[43]. A *Streptomyces rimosus* strain isolated from agricultural soil in the center of Poland was found to use various chitinous substances, e.g., chitosan and shrimp waste, as nutrient sources [19]. Kawase et al. [44] described a *Streptomyces coelicolor* with 13 distinct chitinases: 11 GH18 types (A, B, and C) and two GH19 chitinases. Other actinobacteria have also been found to produce a number of chitinases. For example, *Nocardiopsis prasina* secreted three chitinases, ChiA, ChiB, and ChiBΔ in the presence of chitin [45]. The catalytic domain of the ChiB protein was found to be similar to GH19 chitinases from *Streptomyces* and, as expected with GH19 type chitinases, had high antifungal activity.

As described earlier, *Streptomyces* is one of the most studied genera in terms of chitinase activity, but many other actinobacteria have similar abilities although they are not as well studied. The purified chitinase of *Streptomyces rimosus* exhibited in vitro antifungal properties against *Fusarium solani* and *Alternaria alternata* [19]. Similarly, *Streptomyces viridificans* efficiently lysed the fungal cell walls of *Rhizoctonia*, *Colletotrichum, Aspergillus, Fusarium, Sclerotinia, Curvularia*, and *Pythium* in vitro [30]. Among the *Streptomyces* species isolated from rhizosphere soils, [46] found that *S. hygroscopicus* was antagonistic towards *Colletotrichum gloeosporioides* and *Sclerotium rolfsii*. Culture filtrates obtained at a growth phase when chitinase and β-1,3-glucanase production were highest were the most effective fungal inhibitors.

Actinomycete isolates from soil samples in Jordan exhibited in vitro fungicidal activity against mycelial growth and sclerotia formation of *Sclerotinia sclerotiorum*, when they synthesized chitinase. However, isolates that did not produce chitinase showed only a fungistatic effect [47]. Purified GH18 chitinase from *Streptomyces roseolus* had a marked inhibitory effect on fungal hyphal extensions [32].

Two chitinolytic streptomycetes showed in vitro inhibitory effect on mycelial growth of *Rhizoctonia solani*, which upon infection elicits sugar beet damping-off disease. Soil treatment with either isolate inhibited the disease completely, and significantly improved seedling growth in both infected and uninfected conditions. Compared to the controls, all treatments containing bacteria had increased shoot and root dry biomass [48]. Gherbawy and collaborators [49] found seven strains that synthesized GH19 chitinases with antifungal properties against one or more of the following fungi: *Fusarium oxysporum, Pythium aristosporum, Colletotrichum gossypii*, and *Rhizoctonia solani*.

*Streptomyces viridodiasticus* also produced one or more antifungal metabolites that significantly reduced the growth of the pathogen *in vitro*. When living mycelial mats of *Sclerotinia minor* grown in a carbon-free salt solution were inoculated with *S. viridodiasticus* isolates, all three actinobacterial isolates caused extensive hyphal plasmolysis and cell wall lysis. Furthermore, the isolates, individually or in combination, significantly reduced disease incidence under controlled greenhouse conditions [50]. *Streptomyces cavourensis* was found to be a potential biocontrol agent of anthracnose in pepper, mainly due to a combined effect of chitinolytic enzymes and an antifungal compound, 2-furancarboxaldehyde [51].

### 3.2. Firmicutes

*Bacillus thuringiensis* is a well-known biocontrol agent that has been in use for decades for pest control in agriculture and for the control of disease-related insect vectors.

Many *B. thuringiensis* strains that constitutively express chitinase have been described [7],[52]. Hollensteiner et al. [53] isolated *Bacillus thuringiensis* isolates from tomato roots; the isolates exhibited in vitro antifungal activity against *Verticillium* spp. Only the isolates that carried one or two genes encoding putative chitinases inhibited growth. All the isolates possessed genes encoding the antifungal siderophore bacillibactin, and one isolate had a gene encoding the antibiotic zwittermicin A, suggesting that these chitinolytic bacteria possess multiple antifungal mechanisms [53]. Prassana et al. [54] studied a *Brevibacillus laterosporus* that had two chitinolytic enzymes with GH18 domains, but with different C-terminal domains. The latter determine different substrate specificity to enable efficient hydrolysis.

Interestingly, some studies also report a synergistic effect between *Bacillus thuringiensis* endotoxins and chitinases [38],[55], suggesting that mixed formulas consisting of bacterial consortia for inoculation could be more effective than single strains for biocontrol. *B. thuringiensis* synthesizes two chitinases that enhanced the insecticidal activity of Bt crystal protein against larvae of *Spodoptera exigua* and *Helicoverpa armigera* and almost completely inhibited the germination of *R. solani* and *B. cinerea* spores [7],[56].

Li et al. [57] isolated *Bacillus cereus* from the rhizosphere of eggplant. Using bacterial suspensions, supernatants, and a diluted chitinase solution, these researchers found that all three effectively suppressed germination of fungal spores. In greenhouse experiments, the supernatant and the purified enzymes were less effective than an application of a suspension of the strain's cells in reducing the severity of *Verticillium* wilt on eggplant; the cell suspension reduced the symptoms by 70% in 14 days.

Another firmicute, *Bacillus pumilus*, also was shown to have excellent chitinolytic activity. It was effective not only against several genera of pathogenic fungi of agronomical importance, but it also inhibited the growth of *Scirpophaga incertulas*, a rice pest [58].

*Paenibacillus illinoisensis* isolated from coastal soil in Korea was reported to have strong in vitro chitinolytic activity when assayed on colloidal chitin. It also deformed and destroyed the eggshell of the root-knot nematode (*Meloidogyne incognita*) [59]. Singh et al. [60] found that *Paenibacillus* sp. D1, which was a high producer of chitinase, could be used to control *Helicoverpa armigera*. The use of this strain resulted in a 40% mortality of the larvae, and when combined with acephate (a pesticide), a synergistic effect was observed.

### 3.3. Proteobacteria

In addition to microbes mentioned earlier, numerous other proteobacteria are positive biocontrol agents and have been studied in depth.

The chitinolytic system of *Serratia marescens* has been extensively studied [28]. *S. marescens* produces several GH18 chitinases—ChiA, ChiB, ChiC1, and ChiC2—the latter resulting from a post-translational modification of ChiC1 [8],[61],[62]. These enzymes have different mechanisms of action, which allow for more efficient hydrolysis of chitin [62].

Purified endochitinase and chitobiase of *Serratia marescens* inhibited *Botrytis cinerea* conidiospores and distorted germ tube development in vitro. Prodigiosin, the red pigment produced by *S. marescens*, enhanced inhibition when added together with chitinases [63]. This pigment is also involved in the control of damping-off disease of cucumber, which is caused by *Phytophthora capsici*. Mutants that were defective in prodigiosin synthesis did not have a significant effect on disease symptoms [64].

Strains from the genus *Enterobacter* also inhibited the growth of certain pathogens. Characterized and selected by their chitinase production, the *Enterobacter* spp. were assayed against the common cocoa leaf pathogen, *Colletotrichum gloeosporioides*. Results in vitro showed that the fungal hyphae grew in aberrant shapes and were broken or lysed, which was most likely caused by chitinase activity. In vivo assays demonstrated a decreased severity of the disease [65].

Among the bacteria that had been isolated from the surface horizon of a brown podzolic soil in Ireland and screened for chitinolytic activity, *Stenotrophomonas* and *Chromobacterium* were found to inhibit egg hatch of the potato cyst nematode (*Globodera rostochiensis*) in vitro and in soil microcosms planted with potato seed tubers [66].

Many chitinolytic bacteria have other plant-growth promoting properties as well. One example is a *Pseudomonas* sp. with in vitro antifungal chitinolytic activity, which was shown to enhance nodulation in chickpea [67]. A GH18 chitinase isolated from another *Pseudomonas* sp., which had high amino acid sequence identity with chitinases of *Serratia marescens*, showed little insecticidal activity towards *Spodoptera litura* larvae, but increased the insecticidal toxicity of *Spodoptera litura* nucleopolyhedrovirus [68].

Other taxa have also been studied for their production of chitinases, but have received much less attention, e.g., *Flavobacterium johnsoniae* described by Kharade and McBride [69]. Of five potential chitinases in this species, the authors studied one in depth that had two GH18 domains and that was secreted by a type IX secretion system.

Certain species interactions, as mentioned earlier for the interactions between chitinases and CBPs from different bacteria [26], play a role in chitinolytic efficiencies. Quorum sensing, which is mediated by N-acyl homoserine lactone (AHL) signaling molecules, often regulates enzyme production in gram-negative bacteria. Multiple families of quorum sensing molecules (e.g., N-butanoyl-L-homoserine lactone (BHL) and N-hexanoyl-L-homoserine lactone (HHL)) interactively regulate gene expression in *P. aeruginosa* [70]. In *Chromobacterium violaceum*, HHL mutants were defective in hydrolyzing colloidal chitin when grown in a minimal medium containing the polymer, but could hydrolyze it when the mutant was supplemented with culture supernatant from the wild-type strain or with HHL itself [71]. A chitobiase, now classified as β-N-acetylhexosaminidase, with a high specificity to dimers and also two other β-N-acetylhexosaminidases of 172 and 133 kDa, as well as two endochitinases of 108 and 67 kDa, and a chitobiosidase of 56 kDa were detected by SDS-PAGE [71].

## 4. Uses of Chitinolytic Microbes for Postharvest Disease Control in Crops

Although biocontrol in the field is important to avoid crop loses because postharvest disease has a large economical impact, the use of many common pesticides causes serious health problems. Thus it is important to explore new alternatives for postharvest disease control that reduce economic loss and have no negative effects on human health. Other important factors to be considered are the emergence of resistances by the pathogens and the stringent regulation of pesticide use and disposal, which leads to the need for new solutions to these problems [72].

### 4.1. Direct methods

Chitinolytic microorganisms have been used as biocontrol agents for several crops with promising results. Postharvest diseases have major economic consequences on fruit production. Other crops are equally sensitive to pathogenic fungi and insects upon being transported or stored. The need for alternatives to chemical pesticides is critical towards overcoming postharvest disease.

*Bacillus* spp. that produce chitinases have been used as postharvest biocontrol agents. *Bacillus subtilis* isolates with chitinase activity yielded up to 83% inhibition of *Fusarium oxysporum* and *Botryodiplodia theobromae* infection in yam. Scanning electron microscopy confirmed the complete lysis of the fungal cell wall after 36 hours. The breakdown of the cell walls was hypothesized to be caused by chitinase production [73]. *B. subtilis* strain J9 was suggested to control *B. cinerea* on strawberry under postharvest conditions as a result of its effective chitinase production [74]. Another example is *Bacillus cereus*, which can augement its chitinase expression up to 46.9%, resulting in a reduction of disease in peach caused by *Rhizopus stolonifer*. Peach is a particularly difficult fruit to store because of its high sugar levels and sensitivity to postharvest diseases [75].

### 4.2. Indirect methods

Besides direct inhibition of the postharvest pathogens by chitinase-synthesizing microbes, some organisms cause defense responses via indirect mechanisms, i.e. by inducing the plant to activate its own defense program. Various pathogen-related (PR) proteins, β-1,3-glucanase and chitinases, have been found to play an important part in plant defense against pathogens, and are associated with the systemic acquired resistance response in plants [9]. For example, chitinase production is often enhanced in fruits. Zhang et al. [76] found that apple chitinase production and other defense-related responses were augmented when the stored fruit was inoculated with *Streptomyces rochei* A-1 and challenged with the pathogen *Botryosphaeria dothidea*.

Knowledge about biocontrol has also been applied to transgenic plants, where expression of two combined PR genes, one of them coding for a chitinase, conferred a higher level of resistance than the expression of a single gene. In particular, transgenic carrot, expressing PR-3, which codes for a chitinase, together with expression of a β-1,3-glucanase gene, was found to confer resistance against several pathogens [77].

### 4.3. Chitin soil amendment

Besides the methods that could substitute for or reduce the use of chemicals for pest and pathogen control described above, other options, based on the responses of the soil microbiome, are available. Soil amendment with chitin and its effects have been studied for a long time. Buxton and colleagues [78] found that chitin amendments decreased the severity of disease caused by *Fusarium oxysporum* in a pea field. The *F. oxysporum* population decreased after the amendment, whereas the number of Actinobacteria increased. Several more recent studies show that amending soil with chitin, which selects for the chitinolytic bacteria in the soil resulting in an increase of their numbers, prevents disease. Cretoiu et al. [79], who investigated the long-term effect of chitin amendments in agricultural soil where potato, lily, and wheat were sown during the experiment, showed that fields amended with chitin suppressed the growth of *Verticillium dahliae* and *Pratylenchus* sp. Their abundance decreased 10 times compared to the unamended soil. This suppression could be related to the 10-fold increase in the microbial density of the soil, in particular, the density of chitinolytic bacteria. Enriching the numbers of chitinolytic bacteria to increase the population of biocontrol agents against chitin-containing plant pathogens (e.g., fungi and nematodes) would be a fruitful strategy. Other studies that analyzed the effect of chitin soil amendments on microbial abundance support these findings, such as that of Jacquiod et al. [80] who measured the enrichment of two known chitinolytic taxa, actinobacteria and gamma-proteobacteria, after amending soil with chitin.

## 5. Chitinase Increase in the Soil and the Effect in the Ecosystem

We earlier discussed the advantages of using chitinolytic bacteria in agricultural soil instead of chemical pesticides. However, whether or not an increase in chitinases in soil would affect beneficial fungal and insect populations has to be addressed. On this topic, Vázquez and collaborators [82] studied the effect of fungal antagonist inoculations on arbuscular mycorrhizal fungi. In their study, none of the bacterial inoculations caused any decrease in growth of the fungi, although they all produced chitinases. Moreover, chitinase production did not vary whether the inoculants were coinoculated with the fungi or not. Vauramo and associates [82] described the use of chitinase-producing transgenic *Betula pendula*, which did not affect leaf litter microbial composition. Eight chitinase transgenic birch lines were used with similar results, strongly suggesting that an increase in chitinases does not affect soil community.

## 6. Screening for Chitinase Activity

To detect bacterial strains that produce chitinase for use in agriculture or biocontrol means that reliable tests for chitinase activity must be performed. We describe a few of the more commonly used methodologies. Some of the simplest methods include incorporating chitin into a solid medium and then observing the formation of halos around the colonies as a consequence of chitin degradation [83]. A protocol whereby chitosan instead of chitin is added to the medium has also been described [84].

**Figure 2.**
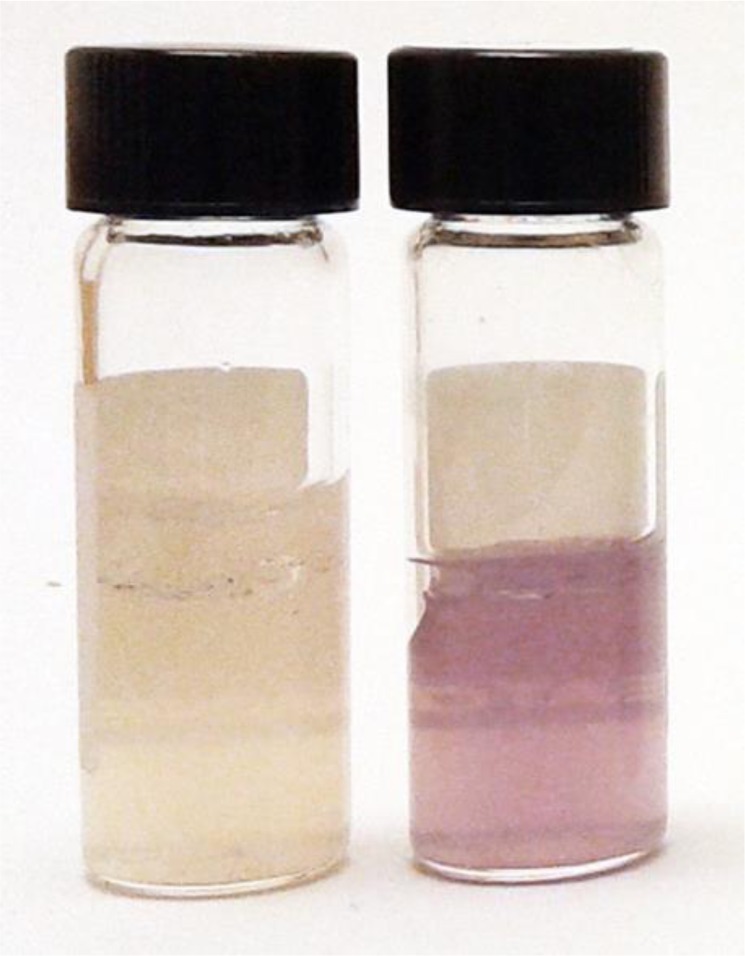
Detecting chitinase activity with a chitin azure colorimetric assay. Chitinase activity can be detected using tubes containing two layers of agar: a top layer consisting of a basal medium with chitin azure, and a lower layer with only the basal medium. When chitin-azure is hydrolyzed in the top layer, the dye diffuses into the lower layer indicating chitinolytic activity. Pictured are two controls used in a chitinase assay, the tube on the left was inoculated with water, while the one on the right was inoculated with a bacterial suspension of a known chitinolytic strain closely related to *Paenibacillus tundrae*.

Various colorimetric assays using chitin azure have also been employed. In our laboratory, we utilized an assay whereby a chitin-azure conjugate is incorporated into a top agar layer containing basal medium containing chitin [85]. If the bacterial strain has chitinase activity, the conjugate breaks down and the disassociated azure dye permeates the bottom agar, which turns purple (Figure 2). Overall, this method yields good results and is an effective first screen, but not all technical or biological replicates exhibit the same degree of color production because chitin is insoluble and its uniform distribution in a culture medium is difficult to attain. Moreover, different concentrations or distributions of the bacteria can also affect the uniformity of the results. Hence, other more analytical methods for detecting chitinase activity should be employed (see review by Howard et al., [86]).

Semi-quantitative or quantitative methods have been developed. For example, Suginta et al. [87] measured chitinase activity by using chitosan labeled with radioactive oligosaccharides, a method, considered by the author to be more accurate than the colorimetric assays. However, additional colorimetric methods have been developed more recently and may be easier and faster to use, and have potential for use in high throughput screening assays [88]. Progress in the techniques for detection, identification, or characterization of chitinases has occurred over the last years, and reviews focusing on the more technical matters have been published. A review article by Duo-Chuan [89] focuses on fungal chitinases and the advances in methodology that have been developed, and screening procedures have been reviewed by Patil et al. [20]. The number of reviews found on only this one topic—technology and methodology of the detection of chitinases—is extensive, thereby highlighting the importance of biotechnology. The use of chitinase and related-enzymes for biotechnological application requires a deep level of understanding, not only of the chitin biochemistry and/or genetics, but also of quantification and accurate detection methodologies of its breakdown products.

## 7. Conclusions

Overall, chitinases are important for pest and pathogen control. Because chitin synthesis is limited to insects, fungi, and some algae, many of which are plant pathogens, this molecule in the pathogen is a logical target for pest control [55]. It is also important to find alternatives to chemical pesticides and to *Bacillus thuringiensis* toxin because resistance to these molecules has already developed [90],[91]. Novel biocontrol agents that serve as alternatives to pesticides, especially those that do not generate resistance and are sustainable and ecologically friendly, have started to appear in the market, and should be a priority goal for the future.

Although chemical pesticides will be used for many years to come, the need for environmentally friendly alternatives is compelling if we are to avoid further damaging the Earth's ecosystems. Chitinolytic microorganisms are a potential alternative to these chemicals because they are already part of the soil and endophytic microbiome, and would thus minimally alter the ecosystem. They also have been found to provide protection against pathogens in several different scenarios, and in some cases, just amending the soil with chitin results in a decrease of pathogens. However, this field of study is still not sufficiently developed and although the findings so far are promising, more research in this direction should be pursued to obtain enough data to bring about an effective solution to the problems facing food production and harvest.
